# Hard Rubber Base

**Published:** 1858-04

**Authors:** Samuel Mallett


### HARD RUBBER BASE.

BY SAMUEL MALLETT.

Messrs. Editors:—With the exception of a single communication to the American Journal, giving the result of my experiments in the use of hard rubber for plates for dental substitutes, I have contented myself with its use in my own practice, choosing to wait until *time*, the great crucible of nature, had proved by actual experience and demonstration, its qualities for becoming a useful servant of the dentist. I have not wished to put my professional brethren to the trouble and expense of adopting a new improvement, merely because I have taken the lead in its development; they have so frequently heard the call, "Lo, here! and lo, there!" that the time has come when all new things should be thoroughly proved, before they are offered for universal adoption.

Having frequently received, since the first announcement of the improvement, letters from my professional brethren and others, regarding the character of the work in various particulars, I have thought it might be of use to state, through the medium of your valuable News Letter, that which may meet the inquiries of various individuals, who are already interested, or may become so, and contemplate adopting it in their practice. And as some may think I have an "axe to grind," I will say here that I have no more *personal* interest in this matter, than any dentist may have who is using it in his private practice. My labor and expense in developing it, I cheerfully give to the profession. It makes no difference to me whether any dentist in the country adopts it or not; but it does make a difference with the public, who are so afflicted as to be under the necessity of resorting to the use of artificial teeth.

The value of the improvement—the value of hard rubber in constructing dental substitutes—I consider now to be settled beyond a question. Although the work has been slowly and cautiously adopted by the profession, and but comparatively few are making use of it, the time will soon come when its merits will be fully known and appreciated.

The universal testimony of those who wear rubber plates, having previously used metal ones, is, that they are much more comfortable and congenial to the mouth than the metal, and *this fact*, other things being equal, must be a ruling considera-tisn in an artificial denture. The manner of forming and packing the moulds was before described. A considerable skill and experience is required to fill the moulds with the prepared rubber, so as to secure success; if the rubber is not packed sufficiently dense, the moulds will not be well filled after vulcanizing; if packed *too* full there will be too great an overflow of the rubber, and a bursting of the mould if it is not very closely confined. From four to five hours are now required to vulcanize a case, or cases, for several may be done at once, as well as one, the number depending entirely upon the size and capacity of the heater. If the case is not in the heater a sufficient length of time, or if the heat has not been raised to, or continued at a sufficient degree, it will come out semi-hard, as it is called, and will prove tender and unfit for use; on the contrary, if it is steamed too much, it will be brittle and of too dark a color. The heat should be raised gradually to about one hundred and seventy degrees, Fahrenheit, and be continued about one and a half hours. It should then be raised to one hundred and eighty degrees, and remain about one hour; at one hundred and ninety degrees, one hour, and from three hundred to three hundred and ten degrees, from one to one and a half hours. The heat is ascertained by a thermometer, which has its bulb inserted in the top of the heater.

Wax plates will be found most convenient for fitting this work in the mouth; the plates should be twice as thick as a gold one, for the purpose of polishing on the lingual side. To prepare the wax in sheets for use, it is rolled with a rolling pin; when it is warm, it may be rolled between two sheets of cotton cloth, or otherwise. If the plate becomes warped in fitting it can be immediately corrected by placing it on the model, or if it has become unequal by pressure, a new one can easily be made.

With this work we are sure of a fit, if the impression be correct, the rubber being steamed to the original model taken from the impression; as the vulcanizing of rubber answers to the fusion of metals, it is the same as if it were cast; every line and irregularity of the model being strongly marked in the plate.

Central cavities should be dispensed with in rubber plates, or if at all used, they should be very small and shallow. There is a fortunate affinity in hard rubber for the mouth, which will enable every practitioner to dispense with this necessary evil, which is found in the use of metal plates.

If the plate is at all approximating to a fair fit, it will hold well after it has been worn a short time, even if it is so imperfect at first, as to be retained in its place only by the aid of the tongue and cheek. The last case which I made having no cavity, required quite an effort with the thumb and fingers to remove it.

Single gum teeth, sections or blocks may be used; those of the former designed for cheoplasty are well adapted to it. I have made use of continuous gums; this may be done by first forming the gum and teeth, and allowing the platina to project about the width of a line all around the edge of the gum for the rubber to close over and hold firmly. This makes beautiful work, but has no advantage over other teeth, as the case will be without seam or joints, whether single or other teeth are used.

The continuous gums require so much platina, that the case becomes heavy and more likely to break by dropping, and then I have feared that the slight elasticity of the rubber would cause the gum to crack, and finally give way, and thus destroy the case.

Rubber plates are very light, so much so, that a case will not sink in water, and *this* feature, it must be conceded is of great value; first, because it enables us to extend the plate and thicken in any manner we choose, for the purpose of restoring the face, without perceptibly increasing the weight of the set. I know of no kind of work which possesses the qualities of accomplishing this object as well as rubber.

Again, this feature of it will be appreciated when we consider the fact, that most accidents occur with teeth out of the mouth, such as dropping, or pressing them against some hard substance. Heavy cases are often broken by falling only two or three inches; rubber cases may be dropped one hundred times on the floor without breaking; it may be thrown across the room with but little danger of injury. With this fact, together with my experience with it in other particulars, I am not at all certain but that rubber plates will prove to be far more *durable* than any other known to the profession. I have not as yet had the first case to repair. If it should be necessary to replace broken teeth, it would be best to make a new plate. The rubber around the fractured tooth *could* be cut out, and the tooth removed, and another tooth fitted in its place, and rubber again steamed around it; but I do not consider that the case will be in as good a condition as it was before the injury; it seems to destroy too much of the life of the rubber to steam it over. The rubber apparently undergoes some chemical change in the act of vulcanization; for this reason, if a failure is made, and it is found that the case is not sufficiently hard and tough, or has not flowed in every part where it was designed that it should flow, it is better to begin anew, rather than to place it in the heater and run it through again.

A few objections have been made to rubber plates, some of them professional and others by non-professional persons; but *all* of them, I think I may say, from men who have not had much, if any actual experience with it themselves. A dentist of high standing averred to me in New York, that he had seen cases that had absorbed the saliva of the mouth, and was considerably disintegrated. I was bound to believe the gentleman, but it must have been caused by the use of poor rubber, or by some imperfection in the manipulation. The plate might have been only semi-hard; I have never seen any thing of the kind in my practice. I have an upper set now before me which has been worn as a temporary for one year, but not the slightest change or appearance of absorption having taken place is perceptible, the plate, by the action of the tongue, only having improved in its polish. The same person said that the plates heated the mouth, the same as a rubber shoe would the foot, by preventing the escape of perspiration. I can only say that nothing like this has ever come under my observation, and I do not think that *hard* rubber can produce this result; the trouble was more likely to have been caused by too large a cavity in the plate, in connection with the natural affinity of the material, causing too much atmospheric pressure. It has been said that block teeth sometimes *break* in steaming. I have only to say to this objection, that I have never known it to occur.

There is not the least taste to the article, which can be easily proved by retaining a piece of it in the mouth for a short time. The sulphuric odor of a case which has not been worn, is entirely destroyed by the saliva. From observation, and the testimony of those who wear them, rubber plates have more of an appearance of cleanliness, and are actually more wholesome than others.

There is but one qualification to be made regarding what I have said about rubber plates, viz: for under sets, when the alveolar process is greatly absorbed, and the muscles are inserted at the very top of the ridge, it may be necessary to use work of greater weight.

The advantages of rubber base, as is seen, are mostly in favor of the patient, and yet I think the dentist will find it more agreeable to insert rubber plates than any other, especially swaged plates. There will be a saving of expense, and also of labor. A rubber plate will not cost any more than a silver one; and further, what is for the interest of the patient, will sooner or later be found to be for the interest of the dentist.

It is not probable that all practitioners of dentistry will run into the use of rubber plates at once; an apparatus will be needed that will cost something; a considerable experience and skill will be required to properly prepare the work, to attend to the boiler and heater; and the whole process must be carried through with carefulness and exactness, or a failure may be expected.—*Dental News Letter.*

